# Physical activity and risk of cancer in middle-aged men

**DOI:** 10.1054/bjoc.2001.2096

**Published:** 2001-09-01

**Authors:** S G Wannamethee, A G Shaper, M Walker

**Affiliations:** Department of Primary Care and Population Science, Royal Free and University College Medical School, London, NW3 2PF, UK

**Keywords:** physical activity, cancer

## Abstract

A prospective study was carried out to examine the relationship between physical activity and incidence of cancers in 7588 men aged 40–59 years with full data on physical activity and without cancer at screening. Physical activity at screening was classified as none/occasional, light, moderate, moderately-vigorous or vigorous. Cancer incidence data were obtained from death certificates, the national Cancer Registration Scheme and self-reporting on follow-up questionnaires of doctor-diagnosed cancer. Cancer (excluding skin cancers) developed in 969 men during mean follow-up of 18.8 years. After adjustment for age, smoking, body mass index, alcohol intake and social class, the risk of total cancers was significantly reduced only in men reporting moderately-vigorous or vigorous activity; no benefit seen at lesser levels. Sporting activity was essential to achieve significant benefit and was associated with a significant dose-response reduction in risk of prostate cancer and upper digestive and stomach cancer. Sporting (vigorous) activity was associated with a significant increase in bladder cancer. No association was seen with colo-rectal cancer. Non-sporting recreational activity showed no association with cancer. Physical activity in middle-aged men is associated with reduced risk of total cancers, prostate cancer, upper digestive and stomach cancer. Moderately-vigorous or vigorous levels involving sporting activities are required to achieve such benefit.   http://www.bjcancer.com © 2001 Cancer Research Campaign


*British Journal of Cancer* (2001) **85**(9), 1311–1316
© 2001 Cancer Research Campaign
doi: 10.1054/ bjoc.2001.2096, available online at http://www.idealibrary.com on IDEAL

http://www.bjcancer.com

### Physical activity and risk of cancer in middle-aged men

**SG Wannamethee, AG Shaper and M Walker**

Department of Primary Care and Population Science, Royal Free and University College Medical School, London NW3 2PF, UK

**Summary** A prospective study was carried out to examine the relationship between physical activity and incidence of cancers in 7588 men aged 40–59 years with full data on physical activity and without cancer at screening. Physical activity at screening was classified as none/occasional, light, moderate, moderately-vigorous or vigorous. Cancer incidence data were obtained from death certificates, the national Cancer Registration Scheme and self-reporting on follow-up questionnaires of doctor-diagnosed cancer. Cancer (excluding skin cancers) developed in 969 men during mean follow-up of 18.8 years. After adjustment for age, smoking, body mass index, alcohol intake and social class, the risk of total cancers was significantly reduced only in men reporting moderately-vigorous or vigorous activity; no benefit seen at lesser levels. Sporting activity was essential to achieve significant benefit and was associated with a significant dose-response reduction in risk of prostate cancer and upper digestive and stomach cancer. Sporting (vigorous) activity was associated with a significant increase in bladder cancer. No association was seen with colo-rectal cancer. Non-sporting recreational activity showed no association with cancer. Physical activity in middle-aged men is associated with reduced risk of total cancers, prostate cancer, upper digestive and stomach cancer. Moderately-vigorous or vigorous levels involving sporting activities are required to achieve such benefit. © 2001 Cancer Research Campaign http://www.bjcancer.com

**Keywords**: physical activity; cancer

There is increasing evidence that physical activity is associated with altered risk of total cancers and certain specific types of cancer, especially colon and prostate (Lee, 1995; Oliveria and Christos, 1997; Gerhardsson, 1997; McTiernan et al, 1998; Moore et al, 1998; Shephard and Shek, 1998). In particular, the evidence strongly supports the role of physical activity in reducing risk of colon cancer (Lee, 1995; Oliveria and Christos, 1997; Gerhardsson, 1997; McTiernan et al, 1998; Moore et al, 1998; Shephard and Shek, 1998; Giovannucci et al, 1995; Slattery et al, 1997); the findings relating to prostate cancer have been inconsistent (Albanes et al, 1989; Thune and Lund, 1994; Oliveria and Lee, 1997; Hartman et al, 1998; Giovannucci et al, 1998; Liu et al, 2000). A few prospective studies have suggested an inverse relationship with lung cancer (Lee and Paffenbarger, 1994; Thune and Lund, 1997; Lee et al, 1999). Data on physical activity and other types of cancer are limited and the amount and type of physical activity required to confer protection remains unclear for any of the cancers. There is some indication that a high level of activity (vigorous) is required to achieve benefit for prostate cancer (Giovannucci et al, 1998). The inconsistent findings between studies for the various cancer types may relate to the different levels of activity in the populations studied. This raises the question of how much activity is required to achieve benefit and in particular, whether light to moderate physical activities have any effect on diminishing risk. This paper examines the relationship between physical activity and the incidence of total cancers and some site-specific cancers and assesses the type and amount of activity required to achieve benefit in a prospective study of middle-aged men.

*Received 9 May 2001*
*Revised 31 July 2001*
*Accepted 2 August 2001*

*Correspondence to:* SG Wannamethee

### SUBJECT AND METHODS

The British Regional Heart Study (BRHS) is a prospective study of cardiovascular disease comprising 7735 men aged 40–59 years selected from the age-sex registers of one group general practice in each of 24 towns in England, Wales and Scotland initially examined in 1978–80. The criteria for selecting the town, the general practice and the subjects as well as the methods of data collection, have been reported (Shaper et al, 1981). Research nurses administered a standard questionnaire that included questions on smoking habits, alcohol intake, physical activity and medical history. Height and weight were measured and body mass index (BMI) defined as height/weight$^2$. The classification of smoking habits, alcohol intake, social class and physical activity have been reported (Shaper et al, 1988, 1991). The men were classified according to their current smoking status: never smoked, ex-cigarette smokers and current smokers at four levels (1–19, 20, 21–39 and ≥ 40 cigarettes/day).

### Physical activity

At screening the men were asked to indicate their usual pattern of physical activity, under the headings of regular walking or cycling, recreational activity and sporting (vigorous) activity. Regular walking and cycling relate to weekday journeys which included travel to and from work. Recreational activity includes gardening, pleasure walking and Do-it-Yourself jobs. Sporting (vigorous) activity includes running, golf, swimming, tennis, sailing and digging. A total physical activity score was calculated for each man based on the frequency and type (intensity) of their physical activities (Shaper et al, 1991). Scores assigned for each type of activity and its duration were based on the intensity and energy demands of the activities reported (Wilson et al, 1986; Taylor et al, 1978). Physical activity at work was excluded from the score because few middle-aged men do physically demanding work and because such activities are not readily amenable to modification. The total score for each man was a relative measure of how much physical activity had been carried out or energy expended. Complete data were available for 7630 men initially grouped into 6 broad categories based on their total score:

### Physical activity index

1 *Inactive* (score 0–2; *n* = 686). No regular activity.
2 *Occasional* (score 3–5; *n* = 2345). Regular walking or recreational activity only.
3 *Light* (score 6–8; *n* = 1761). More frequent recreational activities; or sporting exercise less than once a week; or regular walking plus some recreational activity.
4 *Moderate* (score 9–12; *n* = 1205). Cycling; or very frequent weekend recreational activities plus regular walking; or sporting activity once a week.
5 *Moderately-vigorous* (score 13–20; *n* = 1120). Sporting activity at least once a week or frequent cycling, plus frequent recreational activities or walking; or frequent sporting activities only.
6 *Vigorous* (score ≥ 21; *n* = 513). Very frequent sporting exercise, or frequent sporting exercise plus other recreational activities.

The use of the physical activity score has been validated using heart rate and $FEV_1$ in men free of pre-existing CHD (Shaper et al, 1991). In the analysis the inactive and occasionally active groups have been combined and five groups are used.

### Follow-up

All men have been followed up for all cause mortality, cardiovascular morbidity and development of cancer from the initial screening in January 1978 – July 1980 to December 1997, a mean follow-up period of 18.8 years (range 17.5–20.0 years) and follow-up has been achieved for 99% of the cohort (Walker et al, 2000). Information on death was collected through the established procedures provided by the National Health Service registers.

### Ascertainment of cancer cases

Cancer cases up to December 1997 were ascertained by means of: (1) death certificates with malignant neoplasms identified as the underlying cause of the death (ICD140–209); (2) Cancer registry: subjects with cancer identified by record linkage between the BRHS cohort and the National Health Service Central Register (NHSCR); and (3) postal questionnaires to surviving members in 1992, 1996 and in 1998. In each survey the men were asked whether a doctor had ever diagnosed cancer and if so, the site and year of diagnosis.

Smoking related cancers were regarded as cancers of the lip, tongue, oral cavity and larynx (ICD codes 140, 141, 143–149), oesophagus (ICD 150), pancreas (ICD157), respiratory tract (ICD 160–163), bladder (ICD 188) and kidney (ICD 189).

### Statistical methods

The Cox proportional hazards model (Cox, 1972) was used to assess the effects of physical activity on the risk of cancer. Person-years for each subject was calculated from the time of screening to the time cancer was first diagnosed, death or 1st January 1998. Physical activity was fitted as a categorical variable for the five groups (none/occasional, light, moderate, moderately-vigorous and vigorous). In some analyses, tests for linear trend for physical activity were assessed by assigning quantitative values (1–5) for the five groups of physical activity and fitting physical activity as a continuous variable rather than as categorical variables. In the adjustment, age and BMI were fitted as continuous variables. Alcohol intake (5 levels; none, occasional, light, moderate and heavy), smoking (5 levels; never, ex-smokers, 1–19, 20 and ≥ 21 cigarettes/day) and social class (7 groups; I, II, III non-manual, III manual, IV, V and Armed Forces) were fitted as categorical variables. Direct standardization was used to obtain age-adjusted rates per 1000 person-years using the study population as the standard.

### RESULTS

In the 7630 men with available data on physical activity there were 929 cases of malignant neoplasms (excluding skin cancers) which were obtained from the cancer registry and death certificates (625 cancer deaths). A further 82 cases were obtained from questionnaire data, bringing the total to 1011 cases. Of these, 42 men were diagnosed with cancer prior to or in the same year as screening and these men have been excluded from analysis. Thus total cancer is based on 969 cases (number of men developing cancer) in 7588 men during a mean follow-up period of 18.8 years including 265 lung cancers, 120 prostate cancers and 135 colo-rectal cancers (Table 1). Cancer at more than one specific site developed in 21 men and these cancers have been included under each of the site-specific cancers.

**Table 1** Distribution of cancer sites in 7588 middle-aged British men during 18.8 years follow-up

| | ICD no. | No. of Cases* |
|---|---|---|
| Total | 140–208+ | 969 |
| Lip, oral cavity | | |
| and pharynx | 140–149 | 21 |
| Oesophagus | 150 | 44 |
| Colo-rectal | 153, 154 | 135 |
| Other lower digestive | 151, 152, 155–159 | 109 |
| Stomach | 151 | 59 |
| Liver | 155 | 16 |
| Pancreas | 157 | 29 |
| Lung | 162 | 265 |
| Other respiratory | 160, 161, 163–165 | 11 |
| Bone, connective | | |
| tissue and breast | 170–171, 175 | 7 |
| Prostate | 185 | 120 |
| Testis | 186 | 10 |
| Urinary tract | | |
| Bladder | 188 | 92 |
| Kidney | 189 | 33 |
| Other and unspecified sites | 190–199 | 79 |
| eye and brain | 190–191 | 28 |
| unspecified | 199 | 43 |
| Lymphatic and haematopoietic | 200–208 | 61 |
| Unknown | | 3 |

*Number of men who developed cancer at the various sites. 21 men had 2 or more site specific cancers.
Unknown: 3 men reported doctor diagnosed cancer on questionnaire but did not specify site.

 © 2001 Cancer Research Campaign

**Table 2** Physical activity and age-adjusted cancer rates/1000 person-years and adjusted relative risks for all cancers (excluding skin cancer) in 7588 middle-aged British men

| Physical activity | No. | Cases | Rates /1000 p-y | Relative risks (95% CI) A | Relative risks (95% CI) B |
|---|---|---|---|---|---|
| None/Occ | 3017 | 424 | 8.4 | 1.00 | 1.00 |
| Light | 1749 | 234 | 7.8 | 0.93 (0.79, 1.09) | 0.95 (0.80, 1.11) |
| Moderate | 1196 | 163 | 8.1 | 0.95 (0.80, 1.14) | 1.04 (0.86, 1.24) |
| Moderate-vigorous | 1113 | 101 | 5.8 | 0.68 (0.54, 0.84) | 0.78 (0.62, 0.97) |
| Vigorous | 513 | 47 | 5.7 | 0.65 (0.44, 0.88) | 0.76 (0.56, 1.04) |
| Test for linear trend | | | | *P* < 0.0001 | *P* = 0.02 |

A = age-adjusted
B = adjusted for age, cigarette smoking, BMI, alcohol intake and social class.

**Table 3** Physical activity and adjusted relative risk of site specific cancers in 7588 middle-aged British men

| Cancer site | | None to Moderate (5962) | Moderate-vig (1113) | Vigorous (513) | Test for trend across the groups |
|---|---|---|---|---|---|
| Lung (*n* = 265) | A | 1.00 | 0.57 (0.37, 0.89)* | 0.58 (0.32, 1.07) | *P* = 0.006 |
| | B | 1.00 | 0.77 (0.49, 1.21) | 0.76 (0.40, 1.43) | *P* = 0.19 |
| Upper digestive (*n* = 65) (oral/oesophagus) | A | 1.00 | 0.27 (0.08, 0.86)* | 0.37 (0.09, 1.52) | *P* = 0.02 |
| | B | 1.00 | 0.31 (0.10, 0.99)* | 0.46 (0.11, 1.90) | *P* = 0.05 |
| Stomach (*n* = 59) | A | 1.00 | 0.32 (0.10, 1.01)+ | 0.43 (0.11, 1.78) | *P* = 0.04 |
| | B | 1.00 | 0.40 (0.12, 1.28) | 0.60 (0.14, 2.47) | *P* = 0.15 |
| 'Combined Upper digestive'(124) | A | 1.00 | 0.31 (0.13, 0.70)* | 0.32 (0.10, 0.99)* | *P* = 0.02 |
| | B | 1.00 | 0.37 (0.16, 0.86)* | 0.42 (0.13, 1.32) | *P* = 0.05 |
| Colorectal (*n* = 135) | A | 1.00 | 0.91 (0.55, 1.51) | 0.96 (0.48, 1.89) | *P* = 0.77 |
| | B | 1.00 | 0.90 (0.54, 1.49) | 0.95 (0.48, 1.88) | *P* = 0.74 |
| Bladder (*n* = 92) | A | 1.00 | 0.82 (0.42, 1.59) | 1.85 (0.98, 3.49)+ | *P* = 0.20 |
| | B | 1.00 | 0.90 (0.45, 1.77) | 2.06 (1.08, 3.95)* | *P* = 0.09 |
| Prostate (*n* = 120) | A | 1.00 | 0.94 (0.55, 1.59) | 0.23 (0.06, 0.94)* | *P* = 0.06 |
| | B | 1.00 | 0.89 (0.51, 1.55) | 0.25 (0.06, 0.99)* | *P* = 0.06 |
| Lymphatic and haematopoetic (*n* = 61) | A | 1.00 | 0.70 (0.32, 1.54) | 0.42 (0.10, 1.74) | *P* = 0.15 |
| | B | 1.00 | 0.73 (0.33, 1.63) | 0.47 (0.11, 1.96) | *P* = 0.21 |

A = age-adjusted
B = adjusted for age, cigarette smoking, BMI, alcohol intake and social class. (* excluding skin cancer).
**P* < 0.05 + *P* = 0.06 #*P* = 0.08.

A significant inverse relationship was seen between physical activity and total cancers, with risk significantly reduced only among men engaged in moderately-vigorous and vigorous levels of physical activity (Table 2). No benefit was seen at lesser degrees of physical activity. The inverse relationship persisted, albeit with some attenuation of benefit, even after adjustment for age, cigarette smoking, BMI, alcohol intake and social class.

### Site-specific cancers

Since none/occasional, light and moderate levels of physical activity showed similar risk of total cancer these groups were combined and used as the reference group in showing the age-adjusted relative risk for the selected site-specific cancers (Table 3). Moderately-vigorous and vigorous levels of activity were both associated with reduced risk of cancer in lung, upper digestive tract, stomach and lymphatic/haematopoietic system although for lymphatic/haematopoietic cancers the trend was not statistically significant, possibly because of the small numbers. In subsequent analyses, upper digestive tract and stomach cancers were combined and this combined group is referred to as 'combined upper digestive tract' cancer. Prostate cancer risk was only significantly lower in those engaged in vigorous levels of activity and the trend was of marginal significance. Additional adjustment for cigarette smoking, BMI, alcohol intake and social class attenuated the reduced risk seen with lung cancer. Adjustment made minor differences to the relationships seen with prostate, combined upper digestive tract, and lymphatic/haematopoietic cancers. Vigorous activity was associated with a significant increase in risk of bladder cancer. Moderately-vigorous or vigorous activity showed no relationship with colo-rectal cancer.

### Types of activity

We examined the effects of specific types of physical activity on risk of cancer, adjusting for age, smoking, BMI, alcohol intake and social class (Table 4).

### *Sporting activity*

Men reporting sporting activity ≥ 1 per month showed a significant reduction in risk of total cancers, 'combined upper digestive tract'

*© 2001 Cancer Research Campaign*

**Table 4** Sporting activity and amount of walking and adjusted relative risk of total cancers and site-specific cancers. Adjusted for age, cigarette smoking, BMI, alcohol intake and social class

| | Total | Lung | Combined upper digestive tract | Colo-rectal | Prostate | Bladder | Lymphatic/ haematopoietic |
|---|---|---|---|---|---|---|---|
| Sporting activity > 1/month | | | | | | | |
| No (5508) | 1.00 | 1.00 | 1.00 | 1.00 | 1.00 | 1.00 | 1.00 |
| Yes (2080) | 0.82 (0.69, 0.96)* | 0.81 (0.58, 1.14) | 0.56 (0.32, 0.96)* | 0.90 (0.60, 1.36) | 0.65 (0.40, 1.06)# | 1.50 (0.95, 2.38)# | 0.59 (0.30, 1.16) |
| Regular Walking (mins/day) | | | | | | | |
| < 20 (5540) | 1.00 | 1.00 | 1.00 | 1.00 | 1.00 | 1.00 | 1.00 |
| 20–40 (974) | 1.05 (0.87, 1.26) | 0.90 (0.62, 1.30) | 0.93 (0.52, 1.63) | 1.25 (0.79, 1.99) | 0.73 (0.31, 1.72) | 1.32 (0.76, 2.29) | 1.20 (0.58, 2.47) |
| 40–60 (786) | 0.96 (0.78, 1.19) | 1.04 (0.70, 1.55) | 1.14 (0.64, 2.00) | 0.74 (0.40, 1.38) | 1.09 (0.49, 2.43) | 0.64 (0.27, 1.47) | 1.38 (0.67, 2.83) |
| > 60 (288) | 0.77 (0.54, 1.12) | 0.77 (0.38, 1.56) | 0.97 (0.39, 2.42) | 0.76 (0.28, 2.07) | 0.36 (0.05, 2.65) | 1.43 (0.57, 3.58) | 1.47 (0.52, 4.15) |

cancers and prostate cancer (marginal significance) compared with those who reported no sporting activity or < 1/month. They also showed a reduction in risk of lung cancer and lymphatic/ haematopoietic cancers although the differences were not statistically significant. Little association was seen with colo-rectal cancer. Sporting activity was associated with an increased risk of bladder cancer (marginal significance).

### *Walking*

Risk of total cancer was reduced only in the small group of men who walked more than an hour a day but the reduction in risk was not statistically significant. The reduced risk was seen for lung, colo-rectal and prostate cancers.

### *Recreational activity*

Recreational activity showed little relationship with total cancer. The adjusted relative risks for low (< 4 h/week), medium (4 h/week) and high levels (> 4 h/week) were 1.00, 0.91 (0.76, 1.09) and 0.92 (0.78, 1.10).

### Frequency of sporting activity and cancer

Those who reported sporting type activities at least once a month were divided according to the number of times they carried these out: less than once a week, once a week and at least twice a week. There was an inverse dose-response relationship between cancer risk and the frequency of sporting activity (Table 5). This was seen for prostate cancer, 'combined upper digestive tract' and to a lesser extent for lung cancer and total cancers. A significant and positive dose-response relationship was seen for bladder cancer.

### DISCUSSION

Our finding that physical activity is associated with a significantly reduced risk of total cancers even after adjustment for other lifestyle factors, is consistent with several other prospective studies (Albanes et al, 1989; Paffenbarger et al, 1987; Kampert et al, 1996). However, few prospective studies have examined the type and level of activity required to achieve benefit. In the present study, only moderately-vigorous and vigorous levels of physical activity involving sporting type activities were associated with reduction in risk of total cancers. Lesser levels of activity conferred no benefit. Regular walking for more than an hour a day was associated with reduction in risk of cancer but the number of men engaged in such activity was too small to allow any firm conclusion. Recreational activity showed no beneficial effect on cancer risk, unlike its effects on coronary heart disease (Shaper et al, 1991).

### Site-specific cancers

### *Colo-rectal cancer*

There is considerable epidemiological evidence that increased physical activity is associated with reduced occurrence of colon cancer (Lee, 1995; Oliveria and Christos, 1997; Gerhardsson, 1997; McTiernan et al, 1998; Moore et al, 1998; Shephard and Shek, 1998; Giovannucci et al, 1995; Slattery et al, 1997), but we found little association between total physical activity or sporting activity with colo-rectal cancer after adjustment for potential confounders. Even when analysis was limited to colon cancer, little association was seen. Other prospective studies have either failed to find an association overall or the association found has

 *© 2001 Cancer Research Campaign*

**Table 5** Frequency of sporting activity and adjusted relative risk of cancer and selected cancer sites

| Sporting activity | All cancer | Lung | Combined upper digestive tract | Prostate | Bladder | Lymphatic |
|---|---|---|---|---|---|---|
| None/<1 month (5508) | 1.00 | 1.00 | 1.00 | 1.00 | 1.00 | 1.00 |
| >1/month and <1 week (394) | 0.88 | 1.09 | 1.05 | 0.98 | 1.05 | 0.29 |
| 1/week (759) | 0.86 | 0.82 | 0.50 | 0.63 | 1.37 | 0.87 |
| ≥ 2week (927) | 0.76 | 0.68 | 0.38 | 0.53 | 1.79 | 0.49 |
| Test for trend with increasing frequency | $P = 0.01$ | NS | $P = 0.01$ | $P = 0.05$ | $P = 0.04$ | NS |

been restricted to sub-groups (Albanes et al, 1989; Lee and Paffenbarger, 1994; Lee et al, 1997; Thune and Lund, 1996). The protective effect of physical activity may be of greater importance in older men (Thune and Lund, 1996). When we examined the relationship between physical activity and colo-rectal cancer in younger (< 50 years at base line) and older men, although the numbers were small, in older men vigorous activity was associated with lower (non-significant) risk compared to the less active (none-moderate) men (RR = 0.57 95% CI 0.18–1.82).

### *Prostate cancer*

The findings with regard to prostate cancer have not been consistent (Albanes et al, 1989; Thune and Lund, 1994; Oliveria and Lee, 1997; Hartman et al, 1998; Giovannucci et al, 1998; Liu et al, 2000). We found physical activity to be associated with reduction in risk but only in the vigorous activity group. Regular sporting type of activity at least once a week was required to achieve this effect. These findings are similar to the male health professional study where lower risk was only seen in men engaged in high levels of vigorous activities (Giovannucci et al, 1998). In Norwegian men aged 19–50 followed for 16.3 years only regular physical training during recreational hours was associated with reduced risk (Thune and Lund, 1994). The NHANES I study found recreational (exercise) but not non-recreational activity to be associated with reduced risk (Albanes et al, 1989). In the Harvard Alumni Study only the extremely active men showed a reduction in risk (RR = 0.56), but the number of men engaged at such level of activity was small and the difference was not statistically significant (Lee and Paffenbarger, 1994). Overall, the findings suggest that regular participation in vigorous activity is required to lower risk of prostate cancer.

### *Lung cancer*

Two prospective studies have shown increased levels of physical activity to be significantly associated with reduced risk of lung cancer, even after adjustment for cigarette smoking (Lee and Paffenbarger, 1994; Thune and Lund, 1997; Lee et al, 1999). However, we did not find a significant inverse relationship after adjustment for smoking and other lifestyle factors although an inverse trend was present. We observed a dose-response relation between increasing frequency of sporting activity and reduced risk but the trend was not statistically significant, possibly due to the small number of men engaged in levels associated with apparent benefit. The NHANES I study found no association between recreational activities and risk of lung cancer (Albanes et al, 1989). In the two cohorts which have found a significant effect, the Norwegian cohort (Thune and Lund, 1997) and the Harvard Alumni Study (Lee and Paffenbarger, 1994; Lee et al, 1999), the men were in general more active than the men participating in the British Regional Heart Study. Levels of activity shown to be associated with benefit were relatively high and a relatively high proportion of the men in their cohort were engaged in such activities.

### *Bladder cancer*

Men who were vigorously active showed significant reduction in risk of total cancers and several site-specific forms of cancer, but they also showed significant increase in risk of bladder cancer.

### *Other cancers*

Little information is available on the relationship between physical activity and other types of cancer. In this study, increased physical activity (sporting activity) has been shown to be associated with a significant reduction in the 'combined upper digestive tract' cancers (oral, oesophagus and stomach). In contrast, the Japanese Hawaiian Cancer study found increased activity to be associated with increased risk of stomach cancer but the results were preliminary (Severson et al, 1989). We also observed lower risk of lymphatic and haematopoietic cancers (non-significant) among men who reported sporting activity.

### *Potential confounders*

The association between physical activity and all cancers appeared to be independent of smoking, alcohol intake and pre-existing disease; we have no information on diet. Obesity is an established risk factor for many kinds of cancer and exercise reduces body fat which may decrease the risk of developing cancer (Lee, 1995; Oliveria and Christos, 1997; Gerhardsson, 1997; McTiernan et al, 1998; Moore et al, 1998; Shephard and Shek, 1998). However, there is evidence from this and other studies that physical activity is related to cancer incidence independent of body mass index (Slattery et al, 1997). While adjustment has been carried out for smoking and other risk factors in multivariate analyses, this process can never entirely remove their effects. However adjustment for smoking and other factors considerably reduced the apparent benefit for lung cancer risk associated with moderately-vigorous and vigorous physical activity, and smoking alone accounted for a major part of this reduction in benefit, suggesting that much of the apparent benefit for lung cancer was due to confounding by smoking. In addition, when we separated all cancers into smoking related cancers ($n = 485$) and non-smoking related cancers ($n = 484$) the reduction in risk was more clearly seen for non-smoking related cancers suggesting that the benefit seen for total cancer is not due to residual confounding by smoking (data not shown).

### Mechanisms

Several biological mechanisms have been proposed which might explain the relationship between physical activity and cancer in general and site-specific cancers (Lee, 1995; Oliveria and Christos, 1997; Gerhardsson, 1997; McTiernan et al, 1998; Moore et al, 1998; Shephard and Shek, 1998). Physical activity is known to effect insulin sensitivity, metabolic hormones and growth factors (Shephard and Shek, 1998), all implicated in cancer development (Weiderpass et al, 1997; Singh and Rubin, 1993). Together with the effects of physical activity on cortisol and prostaglandin concentrations, any or all of these mechanisms may condition a general susceptibility to cancer (Shephard and Shek, 1998). There is considerable evidence of the effects of exercise on the immune system and on its ability to inhibit cancer development although the direction of effect may depend on the type of exercise, its intensity and its duration (Shephard and Shek, 1998). It is also possible that some biological mechanisms are site-specific; e.g. colon (Bennett and Del Tacca, 1975), gastro-intestinal tract (Reddy and Wynder, 1977) and prostate (Hackney et al, 1988).

### PUBLIC HEALTH

There is now substantial evidence that regular physical activity has an effect on the development of cancer in general and on certain site-specific cancers. The fact that sporting activities involving moderately-vigorous or vigorous levels of physical activity are required should not inhibit encouragement of regular moderate physical activity towards this end. To carry out sporting activities of the appropriate intensity and duration, a degree of physical fitness is required that can only be achieved by those who have moved gradually through light and moderate forms of activity to more vigorous sporting activity. Heavy exercise has been reported to increase risk of cancers of lung, colo-rectal and pancreas (American Cancer Society, 1992) and in the present study the risk of bladder cancer was increased in the vigorously active men. However it would seem that the overall benefits of regular moderate physical activity on total cancers and on health in general greatly outweigh the possible adverse risks of vigorous activity on one site-specific cancer.

### Conclusions

Physical activity is associated with reduced risk of all cancers, prostate cancer, upper digestive and stomach cancer and to a lesser extent, lung cancer and lymphatic/haematopoietic cancer. However sporting type activities are required to achieve such benefit. We consider that there is now sufficient evidence to justify the addition of cancer to the list of major chronic disorders likely to have their development postponed or prevented by regular physical activity in leisure time.

### ACKNOWLEDGEMENT

The British Regional Heart Study is a British Heart Foundation Research Group and also receives support from the Department of Health. We thank Professor Richard Begent for his helpful advice and encouragement.

### REFERENCES

Albanes D, Blair A and Taylor PR (1989) Physical activity and risk of cancer in the NHANES I Population. *Am J Public Health* **79**: 744–750

American Cancer Society (1992) Cancer Prevention Study II. American Cancer Society Prospective Study. *Stat Bull Met Ins Comp* **72**: 21–29

Bennett A and Del Tacca M (1992) Prostaglandins in human colonic carcinoma. *Gut* **16**: 409

Cox DR (1972) Regression models and life tables. *J R Stat Soc (B)* **34**: 187–220

Gerhardsson L (1997) Physical activity in the prevention and management of cancer. *World Rev Nutr Diet* **82**: 240–249

Giovannucci E, Ascherio A, Rimm EB, Colditz GA, Stampfer MJ and Willett WC (1995) Physical activity, obesity, and risk for colon cancer and adenoma in men. *Ann Intern Med* **122**: 327–334

Giovannucci E, Leitzmann M, Spegelman D, Rimm EB, Colditz GA, Stampfer MJ and Willett WC (1998). A prospective study of physical activity and prostate cancer in male health professionals. *Cancer Research* **58**: 5117–5122

Hackney AC, Sinning WE and Bruot BC (1988) Reproductive hormonal profiles of endurance-trained and untrained males. *Med Sci Sports Exerc* **20**: 60–65

Hartman TJ, Albanes D, Rautalahti M, Tangrea JA, Virtamo J, Stolzenberg R and Taylor PR (1998) Physical activity and prostate cancer in the Alpha-Tocopherol, Beta-Carotene (ATBC) Cancer Prevention Study (Finland). *Cancer Causes Control* **9**: 11–18

Kampert JB, Blair SN, Barlow CE and Kohl HW (1996) Physical activity, physical fitness and all-cause and cancer mortality: a prospective study of men and women. *Ann Epidemiol* **6**: 452–457

Lee I-M (1995) Exercise and physical health: cancer and immune system. *Research Quarterly for Exercise and Sport* **4**: 286–291

Lee I-M and Paffenbarger RS (1994) Physical activity and its relation to cancer risk: a prospective study of college alumni. *Med Sci Sports Exerc* **26**: 831–837

Lee I-M, Manson JE, Ajanu U, Paffenbarger RS, Hennekens CH and Buring JE (1997) Physical activity and risk of colon cancer: the Physicians' Health Study. *Cancer Causes Control* **8**: 568–574

Lee I-M, Sesso HD and Paffenbarger RS (1999) Physical activity and risk of lung cancer. *Int J Epidemiol* **28**: 620–625

Liu S, Lee I-M, Linson P, Ajani U, Buring JE and Hennekens CH (2000) A prospective study of physical activity and risk of prostate cancer in US physicians. *Int J Epidemiol* **29**: 29–35

McTiernan A, Ulrich C, Slate S and Potter J (1998) Physical activity and cancer etiology: associations and mechanisms. *Cancer Causes Control* **9**: 487–509

Moore MA, Park CB and Tsuda HT (1998) Physical exercise: a pillar for cancer prevention. *Eur J Cancer Prevention* **7**: 177–193

Oliveria SA and Christos PJ (1997) The epidemiology of physical activity and cancer. *Ann N Y Acad Sci* **833**: 79–90

Oliveria SA and Lee I-M (1997) Is exercise beneficial in the prevention of prostate cancer? *Sports Medicine* **23**: 271–278

Paffenbarger RS, Hyde RT and Wing AL (1987) Physical activity and incidence of cancer in diverse populations: a preliminary report. *Am J Clin Nutr* **45**: 312–317

Reddy BS and Wynder EL (1977) Metabolic epidemiology of colon cancer. Fecal bile acids and neutral sterols in colon cancer patients and patients with adenomatous polyps. *Cancer* **39**: 2533–2539

Severson RK, Nomura AM, Grove JS and Stemmerman GN (1989) A prospective analysis of physical activity and cancer. *Am J Epidemiol* **130**: 522–529

Shaper AG, Pocock SJ, Walker M, Cohen NM, Wale CJ and Thomson AG (1981) British Regional Heart Study: cardiovascular risk factors in middle-aged men in 24 towns. *B M J* **283**: 179–186

Shaper AG, Wannamethee G and Walker M (1988) Alcohol and mortality: explaining the U-shaped curve. *Lancet* **ii**: 1268–1273

Shaper AG, Wannamethee G and Weatherall R (1991) Physical activity and ischaemic heart disease in middle-aged British men. *Br Heart J* **66**: 384–394

Shephard RJ and Shek PN (1998) Associations between physical activity and susceptibility to cancer. *Sports Med* **26**: 293–315

Singh P and Rubin N (1993) Insulin like growth factors and binding proteins in colon cancer. *Gastroenterol* **105**: 1218–1237

Slattery ML, Potter J, Caan B, Edwards S, Coates A, MA K-N and Berry TD (1997) Energy balance and colon cancer – beyond physical activity. *Cancer Research* **57**: 75–80

Taylor HL, Jacobs DR, Schucker B, Knudsen J, Leon AS and Debacker G (1978) A questionnaire for the assessment of leisure time physical activities. *J Chronic Dis* **31**: 741–755

Thune I and Lund E (1994) Physical activity and the risk of prostate and testicular cancer: a cohort study of 53,000 Norwegian men. *Cancer Causes Control* **5**: 549–556

Thune I and Lund E (1996) Physical activity and risk of colorectal cancer in men and women. *Br J Cancer* **73**: 1134–1140

Thune I and Lund E (1997) The influence of physical activity on lung-cancer risk: a prospective study of 81516 men and women. *Int J Cancer* **70**: 57–62

Walker M, Shaper AG, Lennon L and Whincup PH (2000) Twenty year follow-up of a cohort study based in general practices in 24 British towns. *J Public Health Med* **22**: 479–485

Weiderpass E, Grindley G, Nyren O, Ekbom A, Persson I and Adami HO (1997) Diabetes mellitus and risk of large bowel cancer. *J Natl Cancer Inst* **89**: 660–661

Wilson PWF, Paffenbarger RS, Morris JN and Havlik RJ (1986) Assessment methods for physical activity and physical fitness in population studies: report of a NHLBI workshop. *Am Heart J* **111**: 1177–1192

*© 2001 Cancer Research Campaign*

## References

[PDF_00493] Albanes D., Blair A., Taylor P. R. (1989). Physical activity and risk of cancer in the NHANES I population.. Am J Public Health.

[PDF_00497] Bennett A., Del Tacca M. (1975). Proceedings: Prostaglandins in human colonic carcinoma.. Gut.

[PDF_00500] Gerhardsson de Verdier M. (1997). Physical activity in the prevention and management of cancer.. World Rev Nutr Diet.

[PDF_00502] Giovannucci E., Ascherio A., Rimm E. B., Colditz G. A., Stampfer M. J., Willett W. C. (1995). Physical activity, obesity, and risk for colon cancer and adenoma in men.. Ann Intern Med.

[PDF_00505] Giovannucci E., Leitzmann M., Spiegelman D., Rimm E. B., Colditz G. A., Stampfer M. J., Willett W. C. (1998). A prospective study of physical activity and prostate cancer in male health professionals.. Cancer Res.

[PDF_00509] Hackney A. C., Sinning W. E., Bruot B. C. (1988). Reproductive hormonal profiles of endurance-trained and untrained males.. Med Sci Sports Exerc.

[PDF_00511] Hartman T. J., Albanes D., Rautalahti M., Tangrea J. A., Virtamo J., Stolzenberg R., Taylor P. R. (1998). Physical activity and prostate cancer in the Alpha-Tocopherol, Beta-Carotene (ATBC) Cancer Prevention Study (Finland).. Cancer Causes Control.

[PDF_00515] Kampert J. B., Blair S. N., Barlow C. E., Kohl H. W. (1996). Physical activity, physical fitness, and all-cause and cancer mortality: a prospective study of men and women.. Ann Epidemiol.

[PDF_00522] Lee I. M., Manson J. E., Ajani U., Paffenbarger R. S., Hennekens C. H., Buring J. E. (1997). Physical activity and risk of colon cancer: the Physicians' Health Study (United States).. Cancer Causes Control.

[PDF_00520] Lee I. M., Paffenbarger R. S. (1994). Physical activity and its relation to cancer risk: a prospective study of college alumni.. Med Sci Sports Exerc.

[PDF_00525] Lee I. M., Sesso H. D., Paffenbarger R. S. (1999). Physical activity and risk of lung cancer.. Int J Epidemiol.

[PDF_00527] Liu S., Lee I. M., Linson P., Ajani U., Buring J. E., Hennekens C. H. (2000). A prospective study of physical activity and risk of prostate cancer in US physicians.. Int J Epidemiol.

[PDF_00530] McTiernan A., Ulrich C., Slate S., Potter J. (1998). Physical activity and cancer etiology: associations and mechanisms.. Cancer Causes Control.

[PDF_00532] Moore M. A., Park C. B., Tsuda H. (1998). Physical exercise: a pillar for cancer prevention?. Eur J Cancer Prev.

[PDF_00534] Oliveria S. A., Christos P. J. (1997). The epidemiology of physical activity and cancer.. Ann N Y Acad Sci.

[PDF_00536] Oliveria S. A., Lee I. M. (1997). Is exercise beneficial in the prevention of prostate cancer?. Sports Med.

[PDF_00538] Paffenbarger R. S., Hyde R. T., Wing A. L. (1987). Physical activity and incidence of cancer in diverse populations: a preliminary report.. Am J Clin Nutr.

[PDF_00540] Reddy B. S., Wynder E. L. (1977). Metabolic epidemiology of colon cancer. Fecal bile acids and neutral sterols in colon cancer patients and patients with adenomatous polyps.. Cancer.

[PDF_00543] Severson R. K., Nomura A. M., Grove J. S., Stemmermann G. N. (1989). A prospective analysis of physical activity and cancer.. Am J Epidemiol.

[PDF_00545] Shaper A. G., Pocock S. J., Walker M., Cohen N. M., Wale C. J., Thomson A. G. (1981). British Regional Heart Study: cardiovascular risk factors in middle-aged men in 24 towns.. Br Med J (Clin Res Ed).

[PDF_00548] Shaper A. G., Wannamethee G., Walker M. (1988). Alcohol and mortality in British men: explaining the U-shaped curve.. Lancet.

[PDF_00550] Shaper A. G., Wannamethee G., Weatherall R. (1991). Physical activity and ischaemic heart disease in middle-aged British men.. Br Heart J.

[PDF_00552] Shephard R. J., Shek P. N. (1998). Associations between physical activity and susceptibility to cancer: possible mechanisms.. Sports Med.

[PDF_00554] Singh P., Rubin N. (1993). Insulinlike growth factors and binding proteins in colon cancer.. Gastroenterology.

[PDF_00556] Slattery M. L., Potter J., Caan B., Edwards S., Coates A., Ma K. N., Berry T. D. (1997). Energy balance and colon cancer--beyond physical activity.. Cancer Res.

[PDF_00559] Taylor H. L., Jacobs D. R., Schucker B., Knudsen J., Leon A. S., Debacker G. (1978). A questionnaire for the assessment of leisure time physical activities.. J Chronic Dis.

[PDF_00565] Thune I., Lund E. (1996). Physical activity and risk of colorectal cancer in men and women.. Br J Cancer.

[PDF_00562] Thune I., Lund E. (1994). Physical activity and the risk of prostate and testicular cancer: a cohort study of 53,000 Norwegian men.. Cancer Causes Control.

[PDF_00567] Thune I., Lund E. (1997). The influence of physical activity on lung-cancer risk: A prospective study of 81,516 men and women.. Int J Cancer.

[PDF_00569] Walker M., Shaper A. G., Lennon L., Whincup P. H. (2000). Twenty year follow-up of a cohort based in general practices in 24 British towns.. J Public Health Med.

[PDF_00572] Weiderpass E., Gridley G., Nyrén O., Ekbom A., Persson I., Adami H. O. (1997). Diabetes mellitus and risk of large bowel cancer.. J Natl Cancer Inst.

[PDF_00574] Wilson P. W., Paffenbarger R. S., Morris J. N., Havlik R. J. (1986). Assessment methods for physical activity and physical fitness in population studies: report of a NHLBI workshop.. Am Heart J.

